# Supplementary material for: Afadin Sorts Different Retinal Neuron Types into Accurate Cellular Layers
Source: bioRxiv. 2024 Dec 25:2024.12.24.630272. Preprint. [Version 1] doi: 10.1101/2024.12.24.630272 (PMC11703203; doi:10.1101/2024.12.24.630272)
Supplement: 1 [file NIHPP2024.12.24.630272V1-supplement-1.pdf]

bioRxiv preprint doi: https://doi.org/10.1101/2024.12.24.630272; this version posted December 25, 2024. The copyright holder for this preprint (which was not certified by peer review) is the author/funder, who has granted bioRxiv a license to display the preprint in perpetuity. It is made available under aCC-BY 4.0 International license.

Supplementary Figure 1

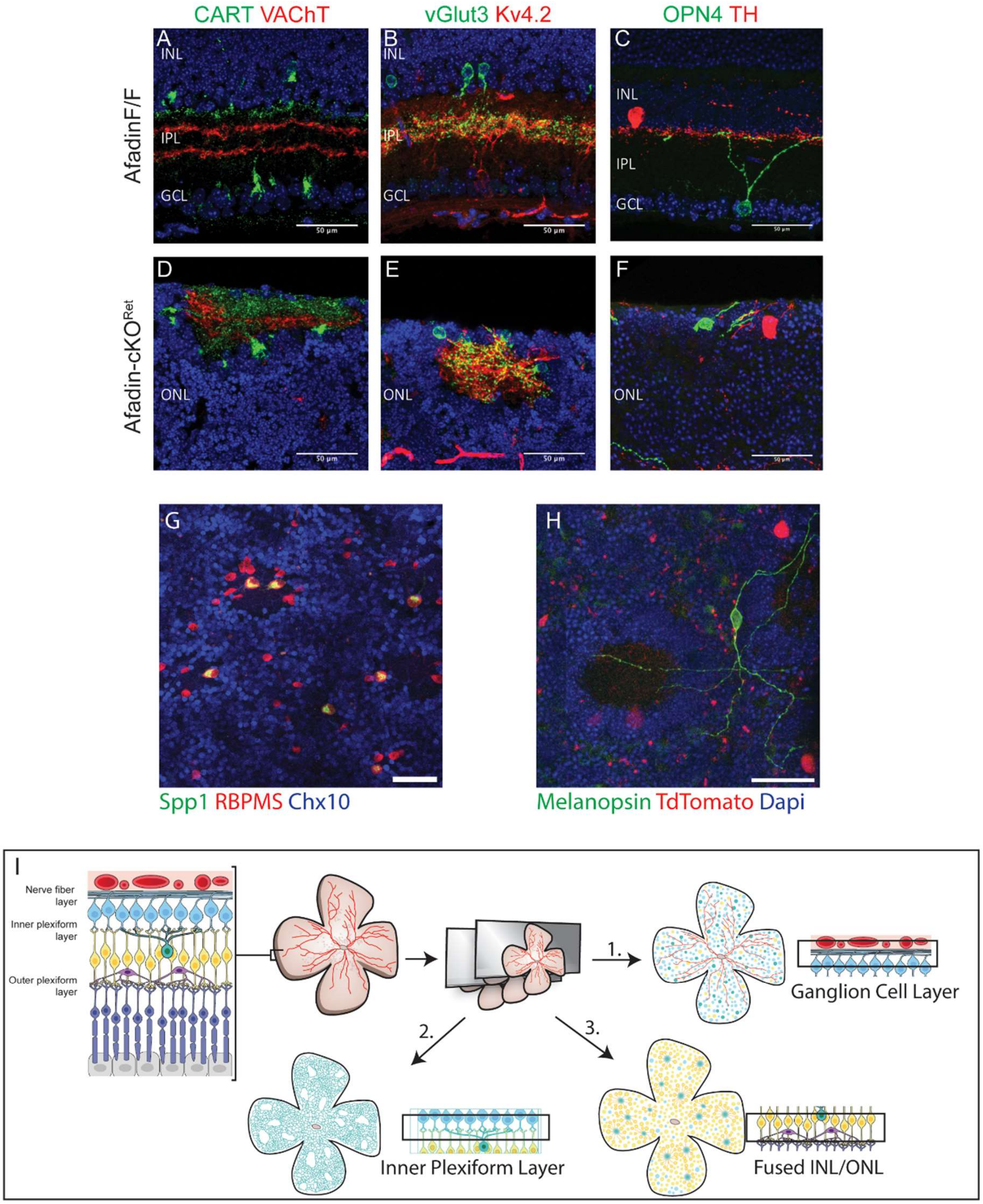

bioRxiv preprint doi: https://doi.org/10.1101/2024.12.24.630272; this version posted December 25, 2024. The copyright holder for this preprint (which was not certified by peer review) is the author/funder, who has granted bioRxiv a license to display the preprint in perpetuity. It is made available under aCC-BY 4.0 International license.

Supplemental Figure 1. Canonical synaptic pairs persist in Afadin-cKO^{Ret} despite mislocalization. (Related to Figure 2).

A-F, Canonical amacrine cell (AC) and retinal ganglion cell (RGC) synaptic pairs in the inner plexiform layer (**A-C**) continue to co-fasciculate in Afadin-cKO^{ret} (**S2D-F**), despite ectopic localization of both subtypes in the outer nuclear layer (ONL). These pairings include ON/OFF direction-selective RGCs (Cartpt) and SACs (VAChT) (**left**), glutamatergic ACs (VGlut3) and S3-IPL-targeting RGCs (Kv4.2) (**center**), and dopaminergic ACs (TH) and intrinsically photosensitive RGCs (ipRGCs)(OPN4) (**right**). Scale bars (A-F): 50 µm.

G-H, In the wholemount view, both αRGCs (Spp1) (**G**) and melanopsin-positive ipRGCs (**H**) were retained near the rosette structures in the fused INL/ONL. Additionally, TdTomato-positive RGCs from AAV-Retrograde injection into the SC are shown (**H**). Scale bars (G-H): 50µm.

I. A diagram illustrating the major findings and wholemount sectioning procedure is shown (**I**).

bioRxiv preprint doi: https://doi.org/10.1101/2024.12.24.630272; this version posted December 25, 2024. The copyright holder for this preprint (which was not certified by peer review) is the author/funder, who has granted bioRxiv a license to display the preprint in perpetuity. It is made available under aCC-BY 4.0 International license.

Supplementary Figure 2

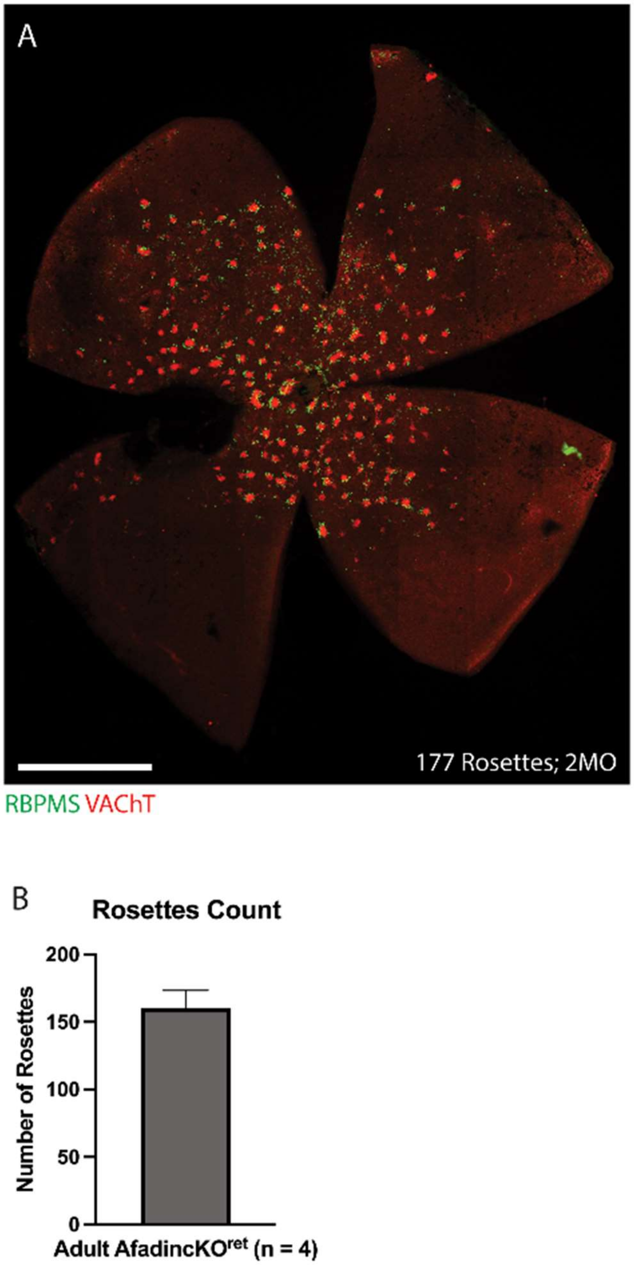

Supplemental Figure 2. Representative Afadin-cKO^{Ret} Wholemount
Representative wholemount displaying rosettes in the fused INL/ONL (**A**): wholemount retina labeled with RBPMS and VAChT aids in the quantification of the number of rosettes per retina. Quantification (**B**) of rosettes across 4 adult Afadin-cKO^{Ret} mice: 160± 13 rosettes across 4 adult Afadin-cKO^{Ret} mice. Data presented as Mean rosette count ± SD.
